# Analysis of the Root Causes of Damage to the Edges of Tank Manholes on the Main Deck of Handy-Size Bulk Carriers

**DOI:** 10.3390/ma14030632

**Published:** 2021-01-29

**Authors:** Leszek Chybowski, Katarzyna Gawdzińska

**Affiliations:** Faculty of Marine Engineering, Maritime University of Szczecin, Wały Chrobrego 1-2, 70-500 Szczecin, Poland; k.gawdzinska@am.szczecin.pl

**Keywords:** hull fracture, inspection manholes, bulk carrier, cracks, corrosion fatigue, steel quality

## Abstract

This study analyzes the root causes of cracks in the deck plating around tank manholes. Four handy-size bulk carriers built in one shipyard were analyzed. In all cases, deck cracks were found near manholes, and the average time from the commencement of operation until the occurrence of cracks was 1356 days. Due to this short wear-life of the vessel’s structural material, the authors believed that it was unlikely to be caused by corrosion fatigue. The authors hypothesized that main decks cracked around manholes because of very poor-quality welded joints and poor-quality steel (large amounts of non-metallic impurities) used to make the manholes. In order to verify this hypothesis, on each of the vessels, material samples were collected from near the cracks and then examined thoroughly. Each sample was subjected to the macroscopic examination of the natural surfaces of cracks and their vicinity, microscopic examination of the material, mechanical property tests, and scanning electron microscope fractography for samples obtained after impact tests. The examination and test results were used to draw detailed conclusions for each case study. The general conclusions based on examination of the whole damage population validated the authors’ hypothesis that main decks cracked around manholes because of very poor-quality welded joints and poor-quality steel used to make the manholes.

## 1. Introduction

Components of ship hulls are subject to variable loads, which result in fatigue and cracking of their structural materials. This problem concerns ships of various types and has existed for many years [1,2]. Since marine vessels are subject to cyclical and time-varying loads and operate in a very corrosive environment (seawater, salty air), it is commonly agreed that ship hull components crack primarily because of corrosion fatigue [3,4,5]. This phenomenon is the result of galvanic corrosion and the variable stresses caused by severe pitting, followed by a transition into cracks filled with corrosion products.

Reliability analysis involves the analysis of physical phenomena that cause the fatigue deterioration of a material and a probabilistic assessment of how the damage may have occurred. The physical aspects of the fatigue destruction of metals have so far been explained using many theories and hypotheses [6], but no comprehensive and coherent theory has exhaustively explained the processes underlying fatigue destruction. This is because the process is very complex, and it is impossible to comprehensively account for all factors. The most important hypotheses of fatigue-related destruction of metals include the following [6,7,8]:

(1) Theories of strengthening and weakening based on experiments conducted by Dehlinger [9] and Gough [10], who showed that strengthening occurs near the glide planes of a crystal lattice under the action of variable loads [11];
(2) Dislocation and vacancy theories are based on the impact of crystal lattice dislocations in metals and interactions [12,13]. These theories were developed by Fujita [14] and Mott [15];
(3) Statistical hypotheses are based on the assumption that the causes of fatigue are in the random (stochastic) material properties. This assumption is based on the fact that every polycrystalline metal is a conglomerate of linked grains containing pores and nonmetallic inclusions [16]. This field was pioneered by Afanasjev [17] and Freudenthal [18];
(4) Energy hypotheses explain how crystal lattice glide bands form slits due to local temperature fluctuations caused by varying loads [8,19]. This hypothesis was proposed by Freudenthal and Weiner [19], and their work was continued by Zakrzewski, Gołaski as well as Oding, [20] and Chowdhury [21];
(5) Decohesion hypotheses assume that a polycrystalline metal contains stress concentrators, such as vacancies, surface irregularities, nonmetallic inclusions, etc. [7]. Fatigue decohesion occurs when the maximum normal stress around a concentrator is equal to the metal cohesive strength. This area was studied by Gołaski, Zakrzewski, Lardner, Bergsmo [22] and others.

There are many corrosion development models for the probabilistic analysis of damage, including probabilistic models invented by Paik et al. [23,24], Akpan et al. [25], Mansour and Wirsching [26], and Downes and Pu [27]. Crack propagation models were developed for a similar approach [28,29,30]. Probabilistic models were approved by the International Maritime Organisation (IMO), allowing the use of the Formal Safety Assessment (FSA) to evaluate a ship’s operational risk [31,32,33]. These models have been progressively developed since 1997. During the reliability analysis of ship hulls, probabilistic methods are commonly used by classification societies [34,35,36].

**Figure 1:**
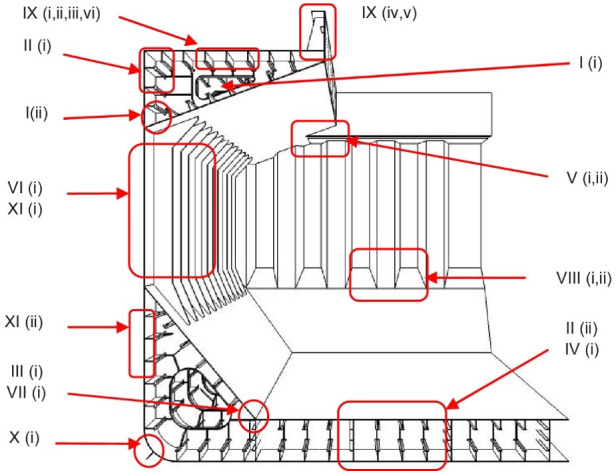
Structural areas prone to fractures in bulk carriers [37].

The bulk carrier structural elements most prone to cracks are shown in Figure 1. According to the classification of the US Ship Structure Committee, these elements are located in area IX (ii)—“Fractures in deck plating around access manholes.” These cracks are the subject of the analysis in this article. Detailed information about all areas presented in the figure is provided in the US Coast Guard document [37].

Because the average time from the start of ship operation until the damage was found was about 1356 days for the four analyzed bulk carriers (it is at least six times faster degradation than in highly loaded energetic machines [38]), it does not appear that this damage was caused by corrosion fatigue.

Because ships were built under the supervision of a renowned classification society, it was assumed that they were designed in accordance with the engineering principles and the society’s requirements. Moreover, it was assumed that the damages were not caused by mistakes in ship operation or maintenance [39], as the observed incidents occurred simultaneously on four different ships operating in different locations around the world.

Nonmetallic inclusions in the material or poor-quality material joints can also reduce the service life of lids [40,41,42]. To determine if this is the cause of the damage, studies must be carried out and the results compared [43]. The authors hypothesized that the main decks cracked around manholes because of very poor-quality welded joints and poor-quality steel used to make manholes, which contain large amounts of non-metallic impurities. These factors were accompanied by excessive mechanical loads associated with hull reactions to waves and operation in a corrosive and low-temperature environment [44,45,46]. Another important factor is the influence of residual stresses resulting from welding on the appearance of cracks inside the welds. This issue was deliberated on in the literature [47,48]. The combination of these factors led to the cracking of the hulls on the examined ships. To analyze the influence of the combination of different factors on a given object failure, decision support tools [49,50] and finite element methods [51,52,53] might be applied.

## 2. Materials and Methods

**Figure 2:**
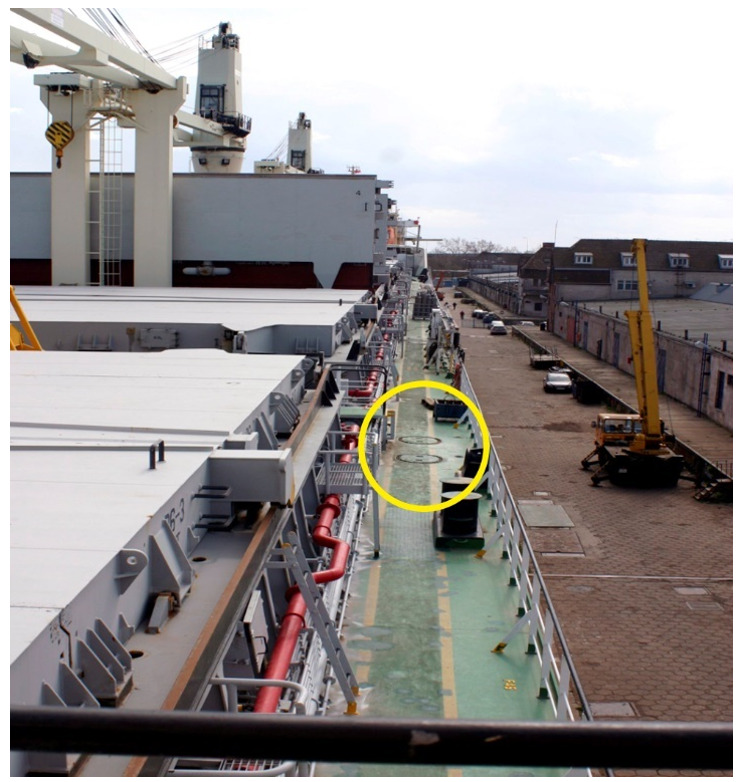
The main deck and tank inspection manhole covers for ballast tanks.

During operation, cracks were found in orifice edges in the deck plating around the access manholes of the ballast tanks. These cracks were examined and tested on-board four handy-size bulk carriers of a major shipowner. Examples of the lids on the main deck of a ship are shown in Figure 2.

All ships were built by the same shipyard from 2011 to 2020, and the basic data of the ships are shown in Table 1.

On each of the analyzed ships in operation, the edges of the holes were found to be cracking in deck plating around access manholes. The estimated times between launch and the examination of causes of the damage to individual ships were 1206 days, 1200 days, 1412 days, and 1606 days, respectively. The time to damage *t* can be described by a two-parameter Weibull distribution, which is universal for various data populations, and its probability density function is

(1) $$f\left(t\right)=\frac{\beta }{\eta }{\left(\frac{t}{\eta }\right)}^{\beta -1}e^{-{\left(\frac{t}{\eta }\right)}^{\beta }}$$

where: *η* is the scale parameter, and *β* is the shape parameter.

**Figure 3:**
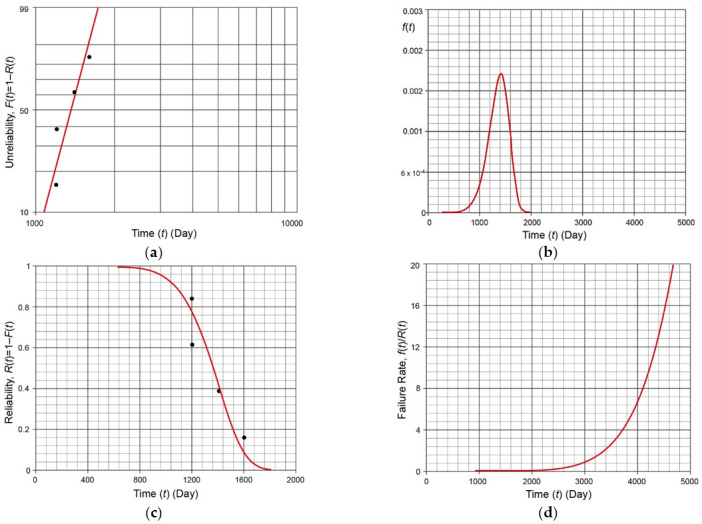
Reliability analysis of observed failures: (**a**) Weibull diagram of the unreliability of the analyzed damage population; (**b**) probability density function of the analyzed damage population; (**c**) the graph of the function of the reliability of the analyzed manholes; and (**d**) the graph of the function of the failure rate for the analyzed manholes.

The analysis carried out with Weibull++ 9.0 (ReliaSoft Synthesis, HBM Prenscia, Tucson, AZ, USA) enabled us to plot the most important reliability characteristics [54,55], shown in Figure 3. For the analyzed population data, the scale parameter was 1429.74 days, and the shape parameter was 7.91.

Preliminary reliability analysis showed that for the analyzed manholes, a crack formed 2000 days after the commencement of the ship operation. The expected lifetime of a ship is usually between about a dozen years and a few decades [56], so if cracks occurred so early into a ship’s operation, the cause must be investigated.

In order to diagnose the root causes of the cracks at the edges of manholes, detailed tests were conducted on each of the ships, including the collection of material samples from around the cracks and carrying out thorough laboratory tests. The chemistry was analyzed using an X-ray microanalyzer. In each of the cases analyzed, the following items were conducted:

(1) A macroscopic visual examination of the natural surfaces of cracks and their surrounding area using a photo camera;
(2) Microscopic examination of materials was performed using optical (including stereoscopic) microscopy. The number of inclusions was evaluated by comparing to patterns in PN-64/H-04510 [57] and PN-66/H-04505 [58];
(3) Mechanical properties examination included static tensile tests and impact tests. Static tensile tests were conducted in accordance with the standard PN-EN 10002 [59]. Impact tests were conducted in accordance with PN-EN 10045-1 [60], PN-EN 10045-2 [61] and PN-79/H-04371 [62]. The standards PN-EN 10045-1 and PN-79/H-04371 are commonly used for this type of testing, and the values given therein do not differ significantly from those required by classification societies;
(4) The fractography analyses were conducted on samples obtained from the impact tests. The morphology of samples with cracked surfaces was examined using a MIRA 3 scanning electron microscope (TESCAN, Brno, Czech Republic) with high-resolution imaging. Components were mapped on the scanned area based on energy-dispersive spectroscopy (EDS, Oxford Instruments, Concord, MA, USA).

For each of the analyzed case studies, specific conclusions were drawn, then presented in the following sections, and used to draw and compile general and final conclusions on the entire ship population.

## 3. Results and Discussion

### 3.1. Case Study 1—Handy-Size Bulk Carrier 30182 DWT

#### 3.1.1. Macroscopic Examination

**Figure 4:**
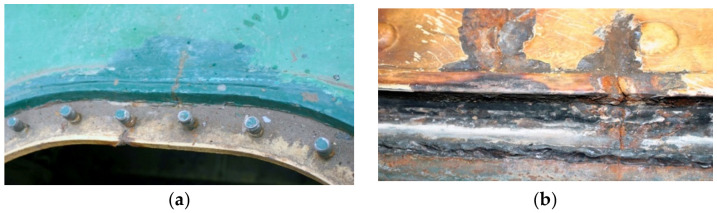
Pictures of origins of cracks: (**a**) view from the outside and (**b**) view from the inside of the tank (handy-size bulk carrier 30182 DWT). Macroscopic examination.

A preliminary macroscopic examination was performed to determine the condition of the cracked material. Figure 4a shows two crack origins in the form of the edges of holes and the edges of the manhole stiffening plate perpendicular to the deck. Examples of both origins of cracking seen from the inside of the tank are shown in Figure 4b.

**Figure 5:**
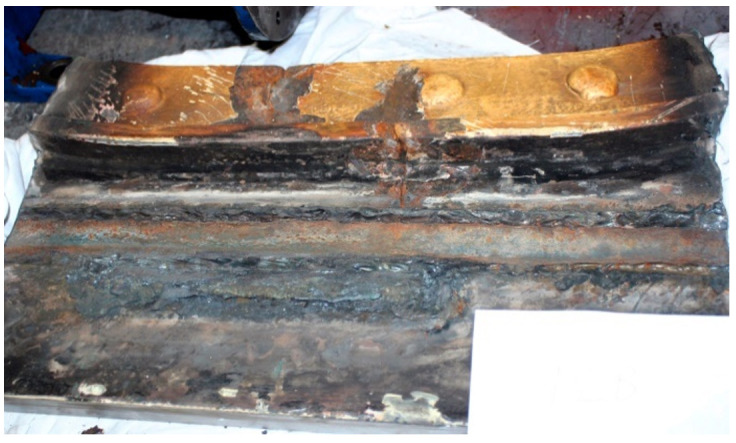
The insert and the welds that join the manhole structure and the deck (handy-size bulk carrier 30182 DWT). Macroscopic examination.

The crack on the right side of Figure 4b developed across a visible weld and then penetrated the insert between the manhole structure and the hole cut out in the deck. The crack does not reach the deck plate (Figure 5), but it is clear that further operation would lead to reaching it.

What contributes to the formation and development of cracks is the very poor quality of weld seams, their number, and the presence of the insert compensating for a hole’s dimensional differences in the deck. There were no cracks in the deck plates, and they were only found in the manhole structure (material examination was limited to the manhole structure).

#### 3.1.2. Microscopic Examination of Materials

**Figure 6:**
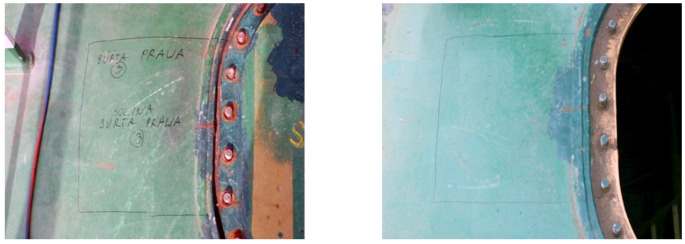
The sections of the manhole structure cut out for examination (handy-size bulk carrier 30182 DWT). Macroscopic examination.

Sections of the manhole structure with cracks were selected and cut out for examination. These locations are shown in Figure 6.

**Figure 7:**
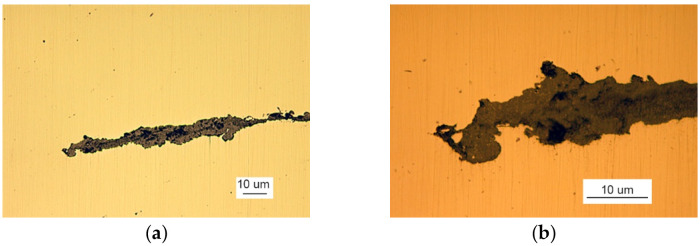
A crack view: (**a**) a crack filled with corrosion products and (**b**) branching crack face (handy-size bulk carrier 30182 DWT). Optical microscopy.

Microscopic examination was performed for the front of the crack that originated at the edge of the manhole. A metallographic specimen was made by merely polishing the piece using diamond pastes with 9 µm and 3 µm grain. Figure 7a shows a crack filled with corrosion products.

Despite the severe corrosion, observation of the crack face makes it possible to conclude that the crack developed along grain boundaries and was branched (Figure 7b).

**Figure 8:**
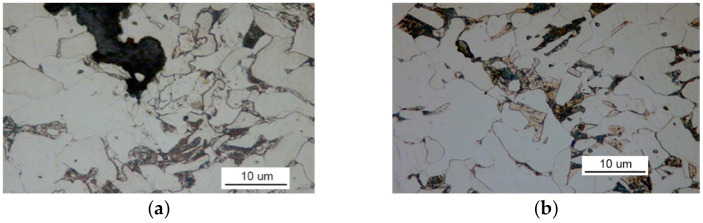
Optical microscopy views: (**a**) the face of the crack after etching with Nital and (**b**) the ferritic and pearlitic structure of the examined material (handy-size bulk carrier 30182 DWT).

After etching the metallographic specimen with nitric acid and alcohol solution, traces of plastic strain followed by the crack front are seen (Figure 8a).

Figure 8b shows that the examined material has a ferrite structure with a small amount of severely-degraded pearlite. At the boundaries of ferrite grains, an intermittent cementite network is visible.

The cracking process can be defined as stress corrosion caused by varying mechanical loads and an aggressive marine environment. The effect of stress within deck plates can be very important under conditions of extreme load in the hull in port or offshore conditions.

#### 3.1.3. Mechanical Property Tests

For the analyzed case study, material samples were tested for their impact strength. The observed material structure containing cementite network precipitates led to testing the steel’s impact strength. Four samples were cut perpendicularly (marking 1) and four parallel (marking 2) to the edge of the manhole opening. The temperature was chosen experimentally to determine the testing range, which were 0 °C, −20 °C, and −40 °C. The results are shown in Table 2.

The material has a significant impact strength at 0 °C. At −20 °C, a large scatter of results is observed, indicating that there is a threshold of brittleness at this very temperature. At −40 °C, the material’s impact strength is low.

#### 3.1.4. Fractography Analysis

**Figure 9:**
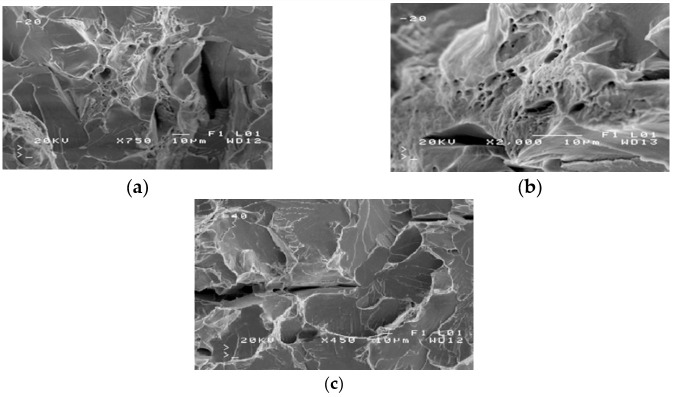
Scanning electron microscope (SEM) views: (**a**) a fracture with a mixed structure.; (**b**) plastic fracture with a crater-like structure; and (**c**) brittle fracture obtained at −40 °C (handy-size bulk carrier 30182 DWT).

The severe corrosion of cracks prevented the fractography analysis of these surfaces. Fractography was performed on the surface of fractures in samples from the impact strength testing at −20 °C and −40 °C. Fractures formed at −20 °C exhibit varied structures. Apart from brittle cracks, along the cleavage planes, there are areas where a crack was preceded by plastic deformation (Figure 9a).

The area of a ductile crack with a crater-like structure is shown in Figure 9b. Figure 9c shows the fractures obtained in tests conducted at −40 °C.

Fractures obtained in tests performed at −40 °C have a brittle structure. Running along cleavage planes, the cracks have a typical “river basin” appearance. Large fracture-based cracks are also observed.

#### 3.1.5. Summary

The analysis of test results for samples taken from handy-size bulk carrier 30182 DWT makes it possible to draw the following conclusions:

(1) Cracks are found in the material of the manhole;
(2) The cracks originated at the edges of manholes and the edges of stiffening plates positioned perpendicular to the deck surface;
(3) The developing cracks crossed the welds and reached the inserts between the hole cut out in the deck and the manhole structure;
(4) The examined cracks occurred because the manhole structure strain under the ship operating conditions exceeded the strength of the steel used.

### 3.2. Case Study 2—Handy-Size Bulk Carrier 30206 DWT

#### 3.2.1. Macroscopic Examination

**Figure 10:**
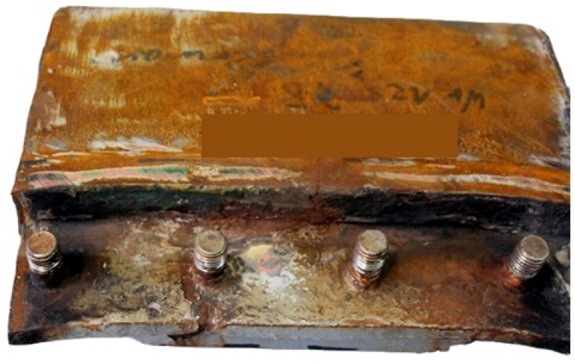
The section of a marine manhole from the handy-size bulk carrier 30206 DWT. Macroscopic examination.

The next tests were performed to establish the causes of cracking in the studied manhole structure using a section of a marine manhole from the handy-size bulk carrier 30206 DWT (Figure 10).

**Figure 11:**
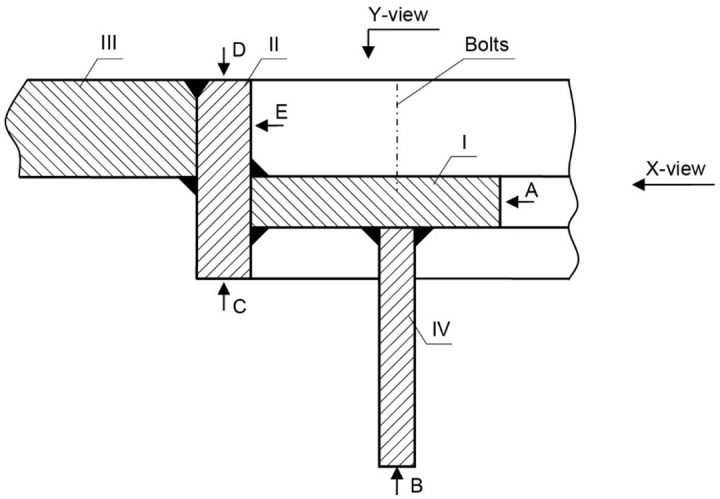
The indication of individual manhole parts from the handy-size bulk carrier 30206 DWT.

**Figure 12:**
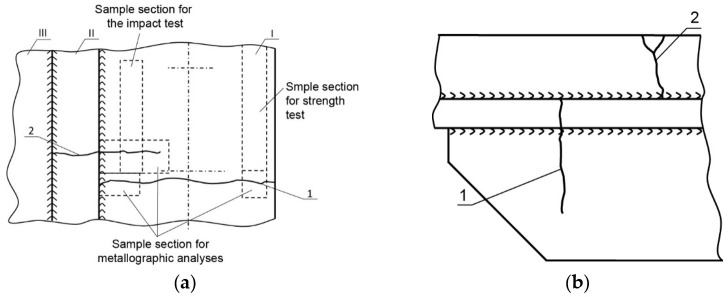
The picture of *y* cracks in a manhole from a handy-size bulk carrier 30206 DWT: (**a**) Y-view and (**b**) X-view.

The manhole structure with the marking of components and their surfaces are shown in the enclosed sketches in Figure 11 and Figure 12. Part I is a component with bolts installed in it, as shown in Figure 1. Part II is a combination of part I with element III, which is the deck named in Figure 10. Part IV is not visible in Figure 10.

**Figure 13:**
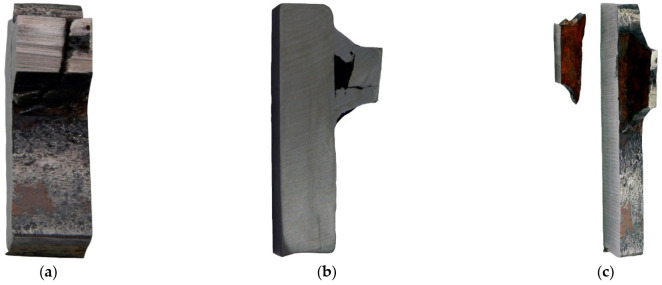
A macroscopic examination of crack 1 (handy-size bulk carrier 30206 DWT): (**a**) the bar cut from elements I and II, containing crack 1; (**b**) the joint between elements I and II and (**c**) the exit of crack 1.

Figure 10 shows two cracks, also numbered 1 and 2 in Figure 12a,b. Crack 1 probably originates on edge A of element I and runs next to the bolt toward element II. Crack 2 originates on edge C of element II (Figure 11). To analyze crack 1, a bar of the plate (12 mm in size, perpendicular to parts I and II) that contained crack 1 was cut out. Figure 13a shows the sample obtained after cutting off a large part of the cracked element I.

A cross section of the joint between the two elements is shown in Figure 13b. The right part in Figure is element II, and the left part is the remainder of the cut-off element I. In the cross-section shown, both elements are virtually not welded together. The joints concerned are similar to two other cross-sections made at a distance of about 100 mm and 120 mm from the first section. In the condition of the area being examined, element I acts independently of the entire manhole structure—they do not form a whole. Because element I is not reinforced by other structural components in this section, it undergoes significant local strain due to the work load. This probably initiates cracking on edge A, although massive corrosion on the fracture surfaces makes it impossible to pinpoint the origin of the crack. The crack extends towards element II, but it does not create another crack since there was no connection between them. It then passes through the weld and exits (Figure 13c).

**Figure 14:**
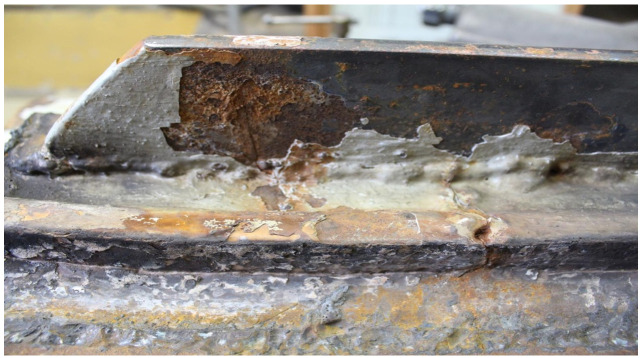
The crack in element IV (handy-size bulk carrier 30206 DWT). Macroscopic examination.

Crack I ends in element I, stays in it, and does not affect the manhole structure, with one exception. The crack is initiated in part IV (Figure 12b), which is welded to part I, and the former reinforces the latter in the plane perpendicular to the deck (Figure 14).

It is very important that element IV is not destroyed in a manner typical for stiffening ribs, in which a crack initiates on its free outer edge as a result of cyclic strain in the plane perpendicular to the deck. Thus, it can be concluded that deformations and the related stresses did not exceed the structure’s ultimate strength in this plane. Element IV was destroyed due to the crack in element I.

**Figure 15:**
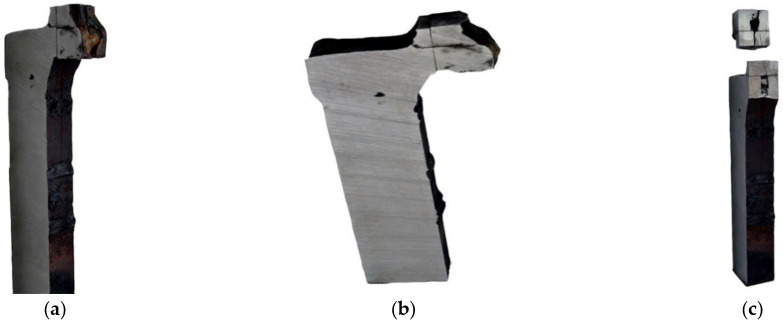
A macroscopic examination of crack 2 (handy-size bulk carrier 30206 DWT): (**a**) the crack running across parts I, II, and III; (**b**) the cross-cut of part II in the area of the origin of crack 2; and (**c**) the cross-cut of part II in the area of the origin of crack 2.

Crack 2 was tested on a 26 mm thick bar containing the analyzed crack, which was cut out perpendicular to edge A of part I (Figure 15a). The right part is element III, and the left part is element II. The figure shows the bottom view of the deck. The crack initiated near surface C of element II. The end view of the bar is shown in Figure 15b. It shows a subsequent section perpendicular to the length of part II and passing through part I parallel to its length. The image of the surfaces obtained from this cut is shown in Figure 15c.

There is a very large metallurgical inclusion, approximately 10 mm in size, in the cross-sectional plane, situated near the origin of the crack. The inclusion is located in the material of part II, near surface C, and caused the initiation of crack 2. The formed crack developed across the entire height of element II up to surface D and reached part III. Next, it ran inside the element over a significant length (Figure 15a) crossing successive welds and spacer pads between the hole excised in the deck and the manhole structure. The crack also developed in element I (Figure 12a).

**Figure 16:**
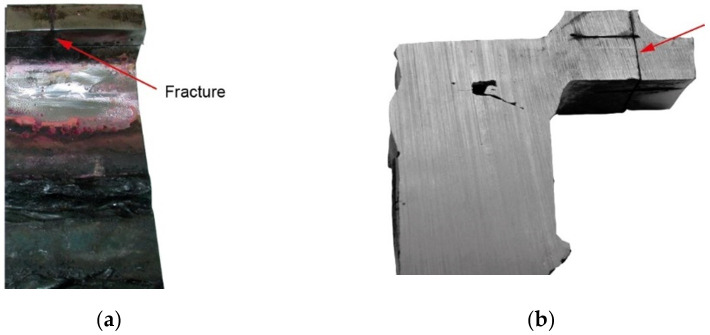
Macroscopic examination of the surface X surroundings (handy-size bulk carrier 30206 DWT): (**a**) the examined bar cut out from parts II and III with a visible crack and (**b**) the lateral surface of the bar cut out (the location of the next cut is shown).

Crack 2 originated in part II next to surface X (Figure 12a) and extended toward part III. To analyze this crack, from part II, a 55 mm wide bar containing this crack was cut out perpendicular to the edge of the manhole. The cut-out bar seen from beneath the deck is shown in Figure 16a, and the cutting surface is shown in Figure 16b. In addition to defects of the welds joining the structural parts of the manhole, another cut near the crack origin was made 12 mm off surface X (Figure 12a). That cut created the surfaces depicted in Figure 15a. At the origin of crack II, a large metallurgical inclusion was found, located on the surface of part II adhered to part 1 and is very close to surface X (Figure 12a).

**Figure 17:**
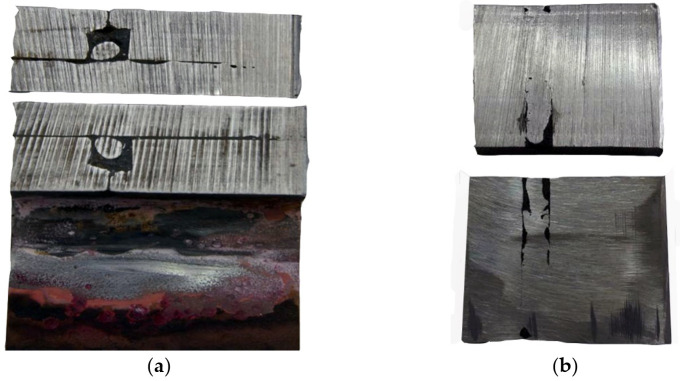
The extensiveness of the inclusion analyzed with the use of macroscopic examination (handy-size bulk carrier 30206 DWT): (**a**) surfaces created by the cut shown in Figure 11 and (**b**) metallurgical inclusion running parallel to the surface of part II.

**Figure 18:**
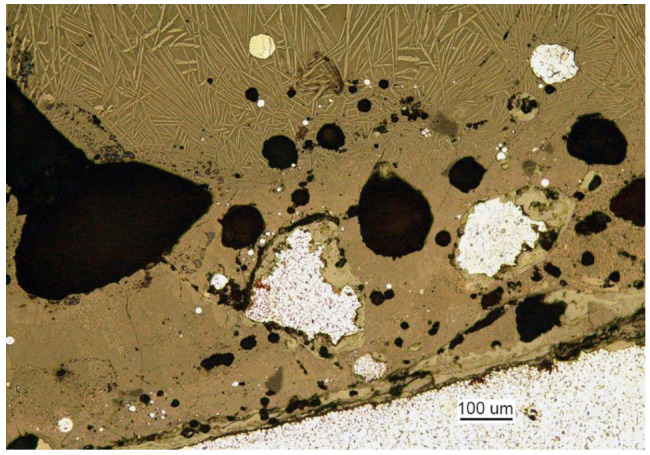
Fragment of the metallurgical inclusion that initiated crack 2 (handy-size bulk carrier 30206 DWT). Stereoscopic optical microscopy.

To show the extensiveness of the inclusion (Figure 17b), another cut was made perpendicular to the surface (Figure 17a), 5 mm off the line between parts I and II. The structure of the inclusion is shown in Figure 18.

X-ray microanalysis of the item was performed using a Hitachi scanning electron microscope (Hitachi, Ltd., Chiyoda, Tokyo, Japan), and the average chemical composition is given in Table 3.

The analyzed inclusion is a portion of the scoured brick lining of a furnace mixed with charge. The analysis shows that the inclusion did not produce a corrosive but rather a mechanical effect that concentrated the stress and thereby initiated the crack and its development. When the crack reached the surface and contacted water, it started to rapidly expand due to stress corrosion that covered a large part of element II but did not reach element III; however, the crack passed through the weld and reached element I.

#### 3.2.2. Microscopic Examination of Materials

**Figure 19:**
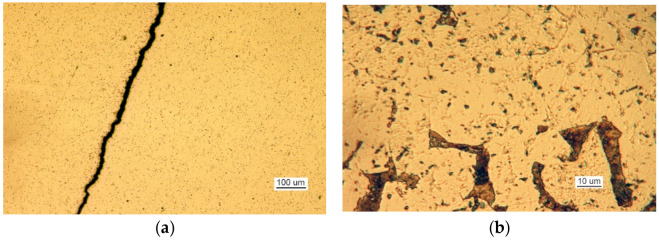
Optical microscopy views (handy-size bulk carrier 30206 DWT): (**a**) the crack and a very large number of spot-wise oxides and carbonitrides. Non-etched specimen; and (**b**) the ferritic and pearlitic structure of the material of element I.

The metallographic examination was conducted on samples cut from elements I and III. A sample of element I was cut from the front of crack 2. The analysis of a non-etched specimen showed a very large amount of nonmetallic inclusions as spots of aluminum oxides and carbonitrides (Figure 19a). There were more oxides than in the TP5a pattern, and the amount of carbonitrides was higher than in the AA5b reference. The examined specimen was etched with an alcoholic solution of nitric acid to reveal the ferritic-pearlitic structure of the steel (Figure 19b). The pearlite has a varied structure with the largest interplate distance corresponding to pattern 7 on scale 1 according to PN-66/H-04505 [58].

**Figure 20:**
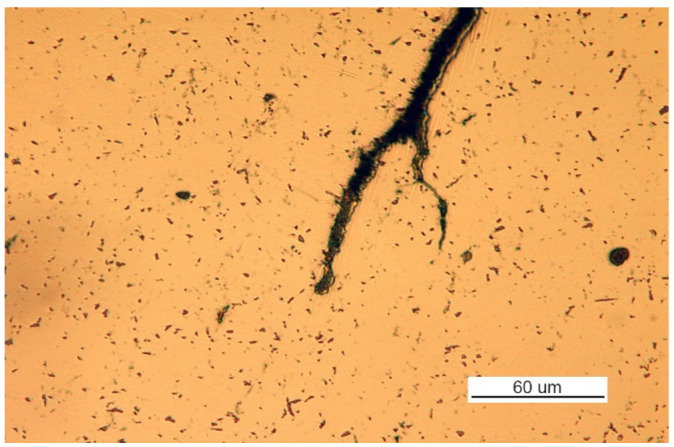
The crack front. Non-etched metallographic specimen (handy-size bulk carrier 30206 DWT). Optical microscopy.

Over its entire length, including the front (Figure 20), the crack was filled with corrosion products, which indicates that cracking was based on stress and corrosion.

**Figure 21:**
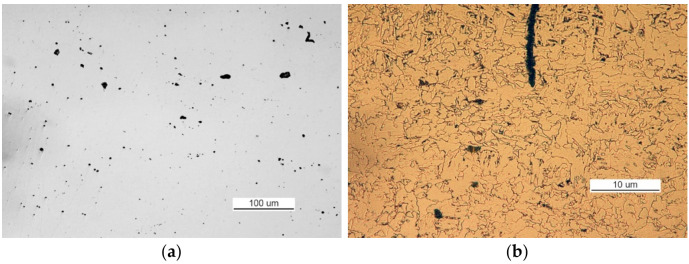
Optical microscopy views (handy-size bulk carrier 30206 DWT): (**a**) nonmetallic inclusions in the structure of element III (non-etched metallographic specimen) and (**b**) structure of the steel near the front of crack 2.

A sample of element III was cut from the front of crack 2. The examination of the non-etched specimen (Figure 21a) shows a large number of nonmetallic inclusions as spot-wise oxides, and this number corresponds to TP5a pattern and globular silicates corresponding to KN5b pattern according to PN-64/H-04510 [57].

The structure of steel in this area is typical for a heat-affected zone, and it contained ferrite supersaturated with carbon and traces of pearlite (Figure 21b).

**Figure 22:**
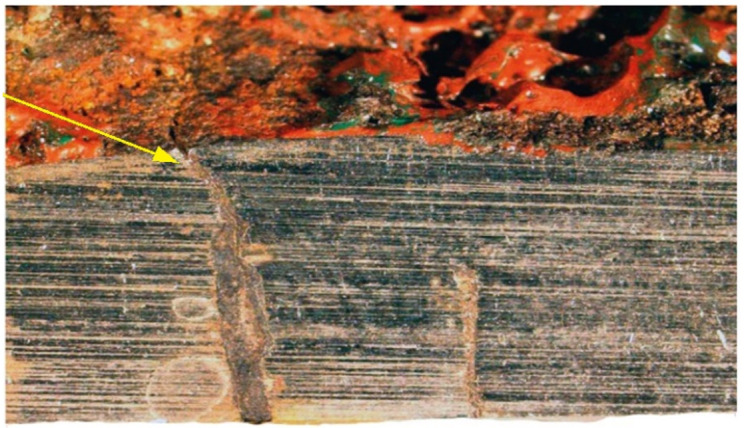
The passage of the crack from part II into part I through the weld that joins the two parts (handy-size bulk carrier 30206 DWT). Macroscopic examination.

Over its entire length (including the front), the crack was filled with corrosion products, which indicates that cracking was based on stress and corrosion. At the origin of the crack, a very large metallurgic inclusion (Figure 22) was found that contained sulfides and other substances. Because the inclusion was located near the surface, water more easily entered the inclusion during vessel operation. In the presence of water, sulfides dissociate to form sulfuric acid; thus, a strong electrolyte is present, creating conditions for intense crack development, indicating that the crack is stress-corrosive.

**Figure 23:**
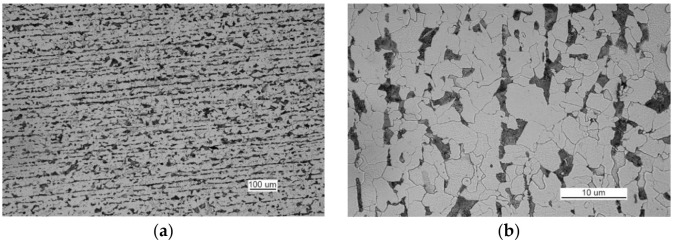
Optical microscopy views (handy-size bulk carrier 30206 DWT): (**a**) the ferritic and pearlitic structure of the steel of part II and (**b**) structure of the steel of part II (the cementite network is intermittent at the grain boundaries).

The structure of the steel used to make part II is ferritic and pearlitic (Figure 23a), with an intermittent cementite network at the ferrite grain boundaries (Figure 23b). The metallographic specimen was etched with an alcoholic solution of nitric acid, and it was observed on a Nikon MM-40 optical microscope (Nikon Instech Co., Ltd., Kawasaki, Kanagawa, Japan).

**Figure 24:**
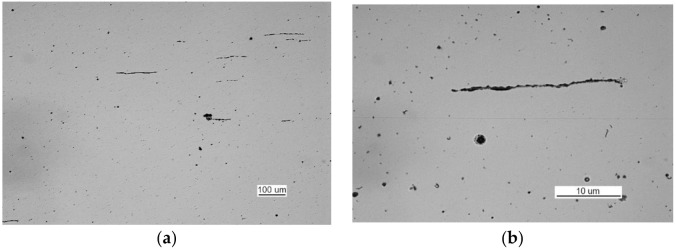
Optical microscopy views (handy-size bulk carrier 30206 DWT): (**a**) numerous rolled-out sulfides and oxide spots and (**b**) rolled-out manganese and iron sulfides with adjacent silicates and numerous oxide spots.

The non-etched specimen exhibited rolled manganese and iron sulfates corresponding to the S3b/4a pattern [57] with adjacent silicates (Figure 24a) and numerous oxide spots (Figure 24b) corresponding to pattern TP3a [57].

#### 3.2.3. Mechanical Property Tests

Static tensile tests were conducted for the analyzed samples. Samples for the strength tests were cut from element I from the location shown in Figure 12a. The results are shown in Table 4.

An impact test was also conducted using samples cut from element I from the location shown in Figure 12a. The results are shown in Table 5.

The test was only conducted at 0 °C, because the impact strength is very low.

#### 3.2.4. Fractography Analysis

**Figure 25:**
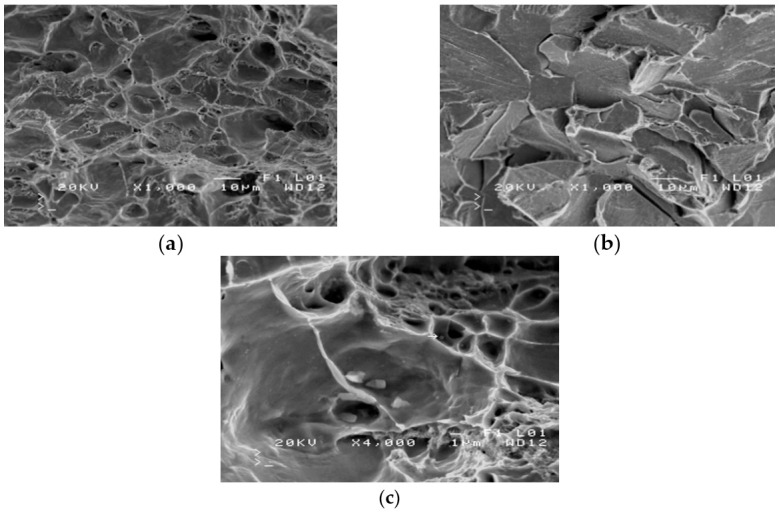
Scanning electron microscope (SEM) views (handy-size bulk carrier 30206 DWT): (**a**) the ductile structure of the fracture; (**b**) the brittle structure of the fracture; and (**c**) nonmetallic inclusions inside craters.

The observed fracture has a mixed structure (Figure 25b,c) that includes ductile, crater-shaped areas (Figure 25a) adjacent to areas of the brittle structure (Figure 25b). There are numerous non-metallic inclusions located inside the craters, as shown in Figure 25c.

#### 3.2.5. Summary

Cracks formed in the examined manhole due to the very poor quality of the welded joints and because poor-quality steel with a large number of non-metallic impurities was used to produce the manhole.

From the condition of element IV, it can be concluded that the loads in the plane perpendicular to the deck did not exceed the strength of the manhole structure. It cannot be concluded from crack 1 that the loads in the plane of the deck exceeded the strength of the manhole structure because only element I was cracked due to the poor quality of its joining with the whole structure. Crack 2 was caused by a metallurgical defect in the material used for element II.

### 3.3. Case Study 3—Handy-Size Bulk Carrier 30210 DWT

#### 3.3.1. Macroscopic Examination

**Figure 26:**
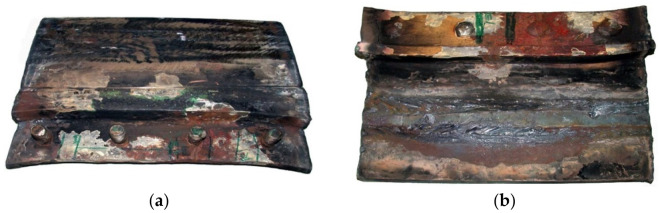
Macroscopic examination results (handy-size bulk carrier 30210 DWT): (**a**) view of a manhole section from the top of the deck and (**b**) view of a manhole section from the bottom of the deck.

Testing was performed to establish the causes of cracking in the examined manhole structure. The testing focused on a section of a manhole supplied from the handy-size bulk carrier 30210 DWT. The section seen from the lid mounting is shown in Figure 26a and from the opposite side in Figure 26b.

**Figure 27:**
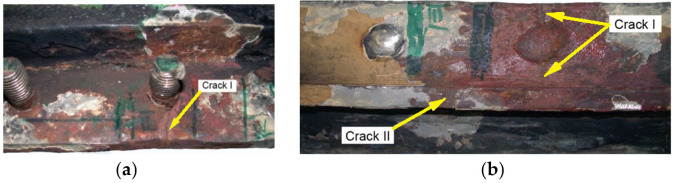
Macroscopic examination (handy-size bulk carrier 30210 DWT): (**a**) view of crack I, (**b**) view of crack II (crack I is visible on the right-hand side).

Two cracks were observed in the examined manhole section. Crack I (visible in Figure 26) is also shown in the close-up photo in Figure 27a, and crack II (visible in Figure 26b) is shown in the close-up photo in Figure 27b.

Crack I in part 1 originates on the edge of the manhole and runs to the bolt and then toward part 2 (Figure 26a). The area near the origin of the crack is corroded, which prevents examination of its structure and the detection of a possible material defect there.

#### 3.3.2. Microscopic Examination of Materials

**Figure 28:**
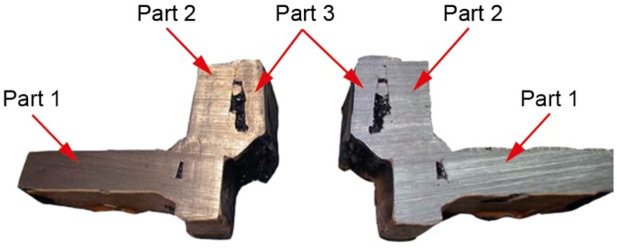
The cross-cut surface. Weld seam defects are shown (handy-size bulk carrier 30210 DWT). Macroscopic examination.

**Figure 29:**
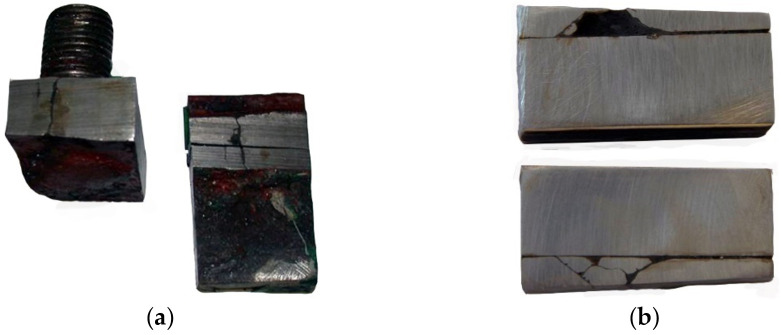
Macroscopic examination (handy-size bulk carrier 30210 DWT): (**a**) crack I and the cutting plane 2-2 and (**b**) the branching end of crack I.

To analyze crack I in part 1, a 32 mm-wide bar containing this crack was cut out perpendicular to the edge of the manhole. The cross-cut surfaces are shown in Figure 28. The sample contains part 1, to which part 2 is perpendicular and the residues of part 3 remain after blasting the manhole structure off the deck. The cross-cut shows the weld joining parts 1 and 2 and the weld joining parts 2 and 3 together with their defects. The excised bar was cut across in plane 1-1 marked in Figure 28, and the resulting cutting plane is shown in Figure 29a.

This last cut created the surfaces depicted in Figure 29b. The branching crack I reaches part 2 and is stopped there.

The analysis of the images obtained from successive cross-cuts indicates that the crack formed on the edge of the manhole in part 1. It then ran towards part 2, and once it reached this part, it branched but did not initiate another crack because the two parts did not contact each other in the cutting plane. The welds joining the two parts are, in fact, tack welds with a relatively small sectional area located on surfaces without full penetration (Figure 28).

**Figure 30:**
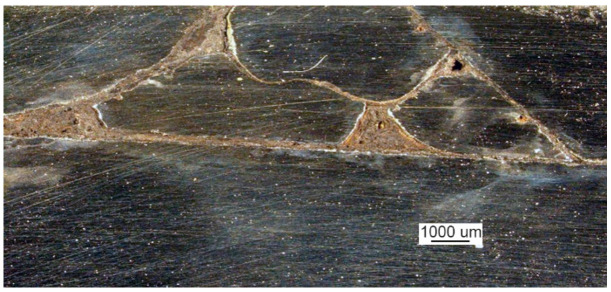
Degree of corrosion of crack I where it branches (handy-size bulk carrier 30210 DWT). Stereoscopic optical microscopy.

Crack I only exists in part 1 and does not cover the entire manhole structure. The crack is corroded along its entire length, and the degree of corrosion here it reaches part 2 is shown in Figure 30.

**Figure 31:**
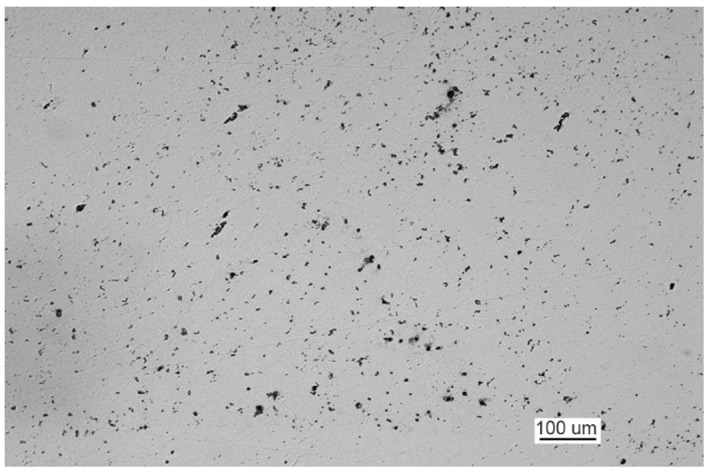
Non-etched metallographic specimen of part 1 showing the presence of spot-wise oxides (handy-size bulk carrier 30210 DWT).

**Figure 32:**
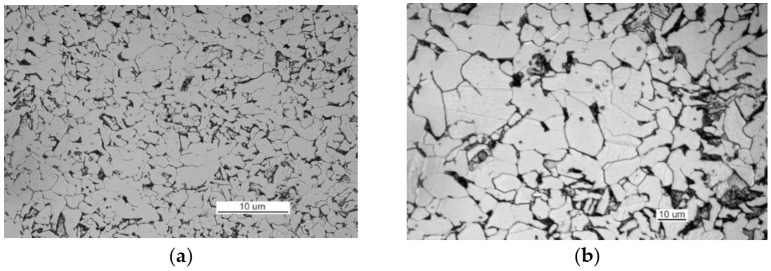
Optical microscopy views (handy-size bulk carrier 30210 DWT): (**a**) the ferritic and pearlitic structure of the material in part 1 and (**b**) the ferritic and pearlitic structure of the material, with visible non-continuous cementite network at the ferrite grain boundaries.

The microscopic analysis of the metallographic specimen of the sample taken from part 1 (Figure 31) revealed large amounts of spot oxides with an intensity exceeding the 5a pattern according to PN-64/H-04510 [57]—Determination of the degree of steel contamination by non-metallic inclusions. After etching with an alcoholic solution of nitric acid, the ferritic-pearlitic structure shown in Figure 32a was obtained.

At the grain boundaries of ferrite, cement was precipitated as a non-continuous network, as illustrated in Figure 32b.

#### 3.3.3. Mechanical Property Tests

Static tensile tests were conducted on two samples with a diameter of ϕ6 mm cut parallel to the edge of part 1 [59], and the results are shown in Table 6.

For condition J2 of this steel, the required impact energy was 27 J, and the result obtained from the impact strength test was only 25% of the required value.

The Charpy impact test was conducted at −20 °C on samples taken from part 1 perpendicular to its edge, and the results are shown in Table 7.

The impact strength test was performed no lower than –20 °C because the obtained impact energy values were very low.

#### 3.3.4. Fractography Analysis

**Figure 33:**
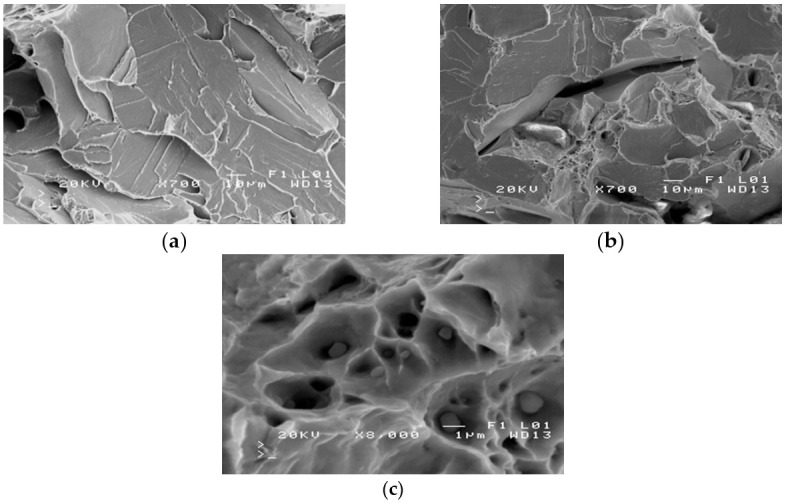
Scanning electron microscope (SEM) views (handy-size bulk carrier 30210 DWT): (**a**) the brittle fracture running along the cleavage planes; (**b**) fracture-based crack and small crater-like areas; and (**c**) spherical precipitates of silicon and aluminum oxides at the bottom of craters.

The results of the fractographic analysis of samples from handy-size bulk carrier 30210 DWT are shown in Figure 33.

The fractographic analyses revealed brittle fracture (Figure 33a) with numerous fracture-based cracks (Figure 33b) and negligible traces of permanent strain as small breaks. There were also a few areas with small craters, the bottom of which contained spherical precipitates of silicon or aluminum oxides (Figure 33c).

#### 3.3.5. Summary

For crack I, deformations of the deck and the associated stresses were transferred to part 1 across the welds joining parts 1 and 2. They are located only on the surface, have no weld penetration, and have relatively small cross sections. Despite this, no cracks were found in these welds except insignificant, negligible cases; thus, it is unclear why part 1 cracked. It had a relatively large cross section. Analysis of crack I indicates that the crack was caused by stress corrosion, which means that the crack was caused by the synergistic action of two factors—mechanical loads and the corrosive environment.

Crack II was initiated by stress concentration caused by a massive metallurgic inclusion located very close to the surface. Its chemical composition indicates that it is a scoured furnace lining element that is not corrosive but only has a mechanical effect. However, after the crack reaches the surface and contacts water, it propagates by stress corrosion in the manner described for crack I. It is highly probable that cracks of this type are mainly caused by excessive tensile loads affecting deck plates as the hull reacts to waves. In contrast, during continued operation, the corrosive environment becomes active after the crack has been formed.

The root cause of cracking in the elements of the examined manhole structure is the use of low-quality steel heavily contaminated by nonmetallic inclusions. The latter include large notches that concentrate working stresses and initiate cracks. All of these inclusions significantly reduce the impact strength (ductility) of the steel to an unacceptably low level. The second cause of cracking in manhole structural elements are poor-quality welded joints and welds without penetration, which create notches that initiate cracks. The effect of stress in the deck plates may be the third cause under the conditions of extreme hull loads in port and offshore conditions.

### 3.4. Case Study 4—Handy-Size Bulk Carrier 30185 DWT

#### 3.4.1. Macroscopic Examination

**Figure 34:**
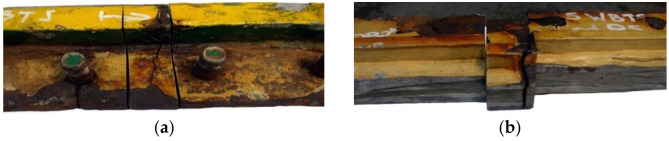
Macroscopic examination (handy-size bulk carrier 30185 DWT): (**a**) lid view of the manhole section marked 5WBTS and (**b**) bottom view of the manhole section marked 5WBTS.

This examination was aimed to determine the causes and circumstances of cracks in the manhole section structure. The delivered test piece is a ship’s manhole section marked 5WBTS. The section seen from the lid mounting is shown in Figure 34a and from the opposite side in Figure 34b.

**Figure 35:**
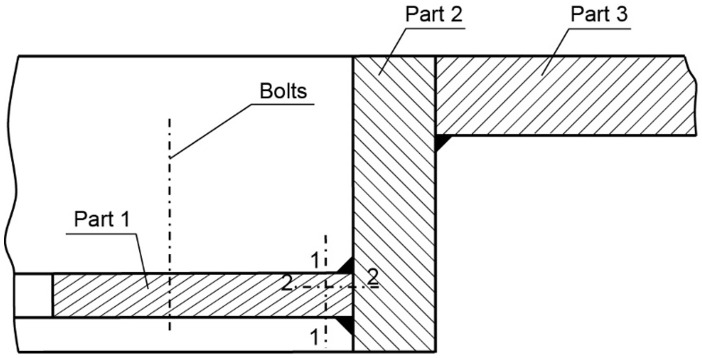
Cross-section diagram of the manhole structure (handy-size bulk carrier 30185 DWT).

Figure 35 shows a cross-section of the manhole structure with the individual parts marked. Part 2 is a combination of part 1 (the screws fitted in it are visible in Figure 34, and part 3 is part of the deck.

**Figure 36:**
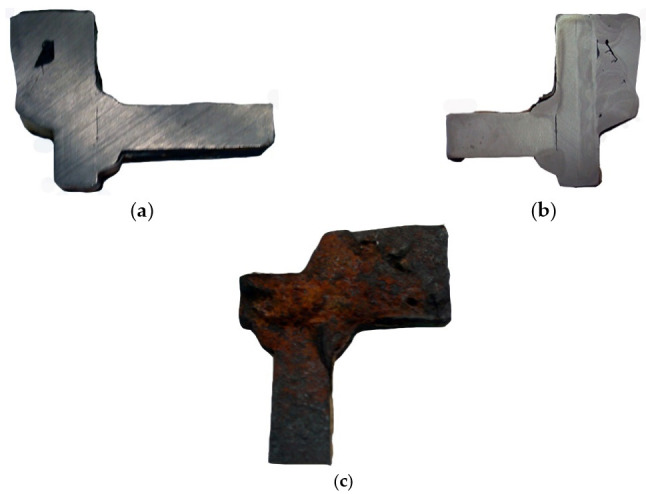
Macroscopic examination (handy-size bulk carrier 30185 DWT): (**a**) the lateral surface of the bar containing crack I; (**b**) view of the second side surface of the bar after etching; and (**c**) fracture surface.

Two cracks were identified in the section shown in Figure 36. The right-hand crack, visible in the cut-out bar, is hereafter referred to as crack I, and the left-hand crack passing through the bolt hole is referred to as crack II. The lateral surface of the bar containing crack I is shown in Figure 36a.

There is a lack of penetration in the joint bonding parts 1 and 2 and a defective joint between parts 2 and 3. The second side surface was sanded and etched (Figure 36b). Defects in the weld seams and corrosion pitting running from the crack surface are visible.

Part 1 was cut 25 mm off the part’s edge (Figure 35, cut 1-1) so that the cracked part of the bar was separated, and the fracture surface was exposed (Figure 36c).

**Figure 37:**
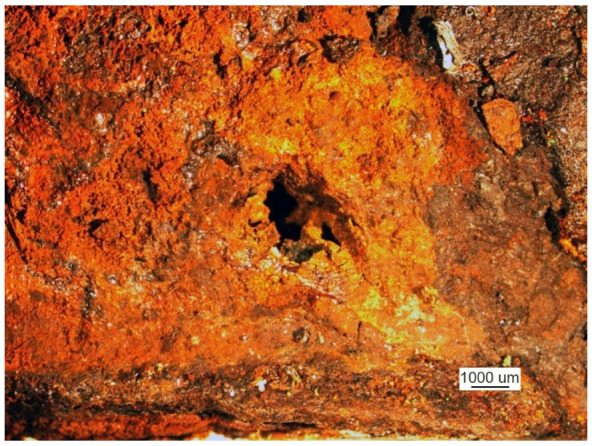
The deep corrosive pit is the presumed origin of the crack (handy-size bulk carrier 30185 DWT). Stereoscopic optical microscopy.

The crack surface was very corroded, making fractographic analysis impossible. Part 2 exhibited only a single large pitting, which probably remained due to local material heterogeneity and, presumably, is the origin of the crack. The view of the pitting under a stereoscopic microscope is shown in Figure 37.

**Figure 38:**
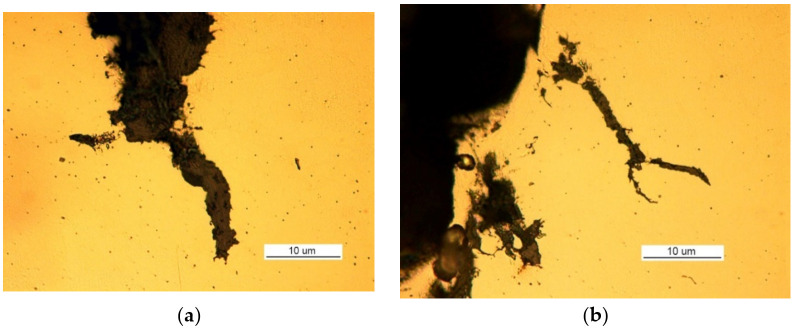
Optical microscopy views (handy-size bulk carrier 30185 DWT): (**a**) the front of the crack developing in part 1 and (**b**) secondary cracks filled with corrosion products.

The crack covered all of part 2 and developed in part 3 (to an unknown depth) and part 1 through both welds that join parts 1 and 3 to part 2. The front of the crack in part 1 was further examined. The sample covering the crack front area was cut parallel to the surface of part 1 at a depth of about 6 mm. The front of the crack is shown in Figure 38a.

Corrosion products were observed within the entire crack, including its front. No part contains a crack that would be caused only by a mechanical load because the branches of the crack, including its face, are filled with corrosion products (Figure 38b).

The results show the important role of corrosion processes in the cracking of materials. The corrosion processes intensify due to corrosive conditions, which can be regarded as being non-variant in the first approximation, and due to the material’s purity and homogeneity. It should be noted that cracks near the edge of the deck manhole can also occur if the permissible stress (i.e., mechanical load) is significantly exceeded in this area.

**Figure 39:**
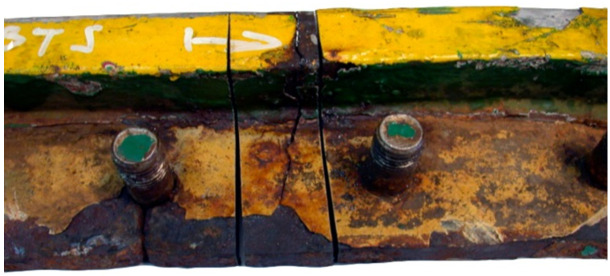
Crack II running through a bolt hole (handy-size bulk carrier 30185 DWT). Macroscopic examination.

Figure 39 shows a close-up photo of a 5WBTS section with crack II running from the edge of part 1 to the hole in which the bolt is placed and then to part 2.

**Figure 40:**
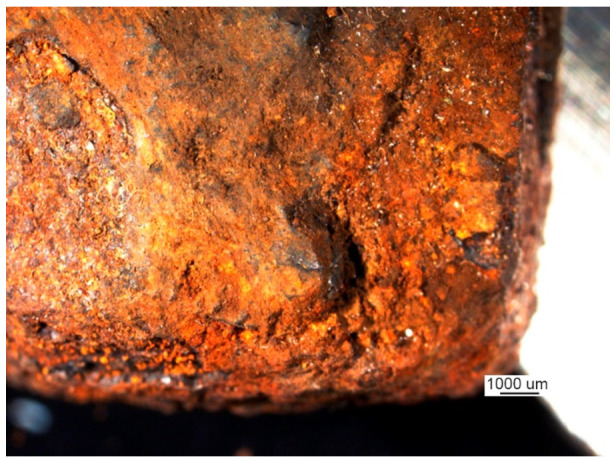
Corrosion pits at the edge of part 1 (handy-size bulk carrier 30185 DWT). Stereoscopic optical microscopy.

The origin of the crack is located near the edge of part 1. The large corrosion cavities near the origin (visible in the figure) make a fractographic analysis of the fracture surface impossible; thus, it is not possible to determine the cause of the crack at this location. It can only be concluded from the numerous and deep corrosion pits located near the edge of part 1 that the origin of the crack was located here because of material heterogeneity due to non-metallic inclusions (Figure 40).

The camera’s relatively small depth of field does not allow the full depiction of the plasticity of the stereoscopic microscope image.

**Figure 41:**
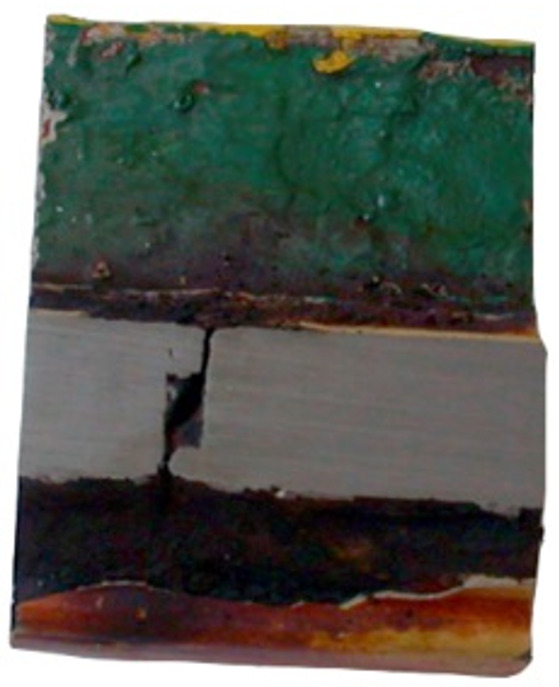
The merging of cracks previously branched at the bolt thread (handy-size bulk carrier 30185 DWT). Macroscopic examination.

The crack expanding across the bolt hole was split at this point into two parallel cracks, which then merged into one crack, what is visible in the cross section made 50 mm from the edge of part 1 (Figure 41).

**Figure 42:**
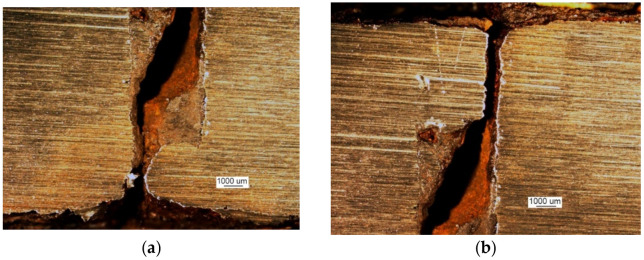
(**a**,**b**) Part of the location where the cracks merged (handy-size bulk carrier 30185 DWT). Stereoscopic optical microscopy.

Figure 42 shows where the cracks merged, which is a special point because it can have one of two natures. It can be either a rolled, large, non-metallic inclusion (as its shape indicates), or a very large number of products of intensive corrosion caused by stress at the crack face.

**Figure 43:**
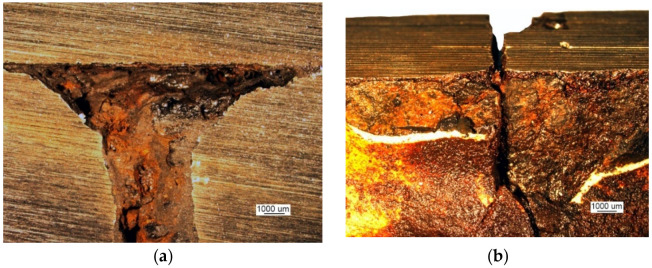
Stereoscopic optical microscopy views (handy-size bulk carrier 30185 DWT): (**a**) crack reaches part 2 and (**b**) crack transition from part 1 (upper part of the figure) to the joint.

The first case confirms the poor quality of the steel used, and the second case proves the effect of stress on the expansion of cracks and the synergistic effect of stress and corrosion. Cut 2-2 (Figure 35) revealed the route of the crack until it reached part 2, where it branched and severe corrosion occurred (Figure 43a). At this point, the crack did not enter part 2 because there is no connection between the parts, but it expanded and penetrated the joint on the surface of both parts (Figure 43b).

#### 3.4.2. Microscopic Examination of Materials

**Figure 44:**
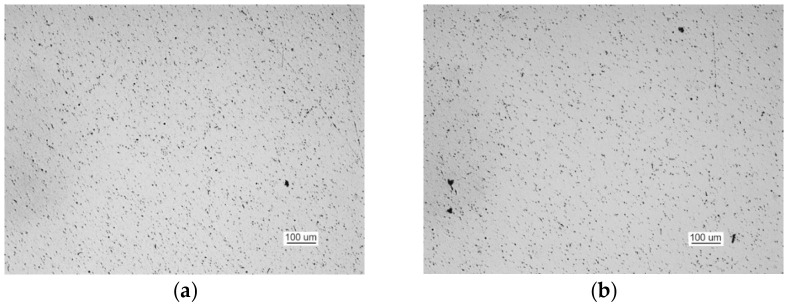
Optical microscopy views (handy-size bulk carrier 30185 DWT): (**a**) oxide spots in the material of part 1 (non-etched metallographic specimen) and (**b**) oxide spots in the material of part 2 (non-etched metallographic specimen).

The examination of non-etched specimens made from samples taken from parts 1 and 2 showed very large amounts of nonmetallic inclusions, mainly oxide spots, which exceed the TP5a pattern [57] (Figure 44).

**Figure 45:**
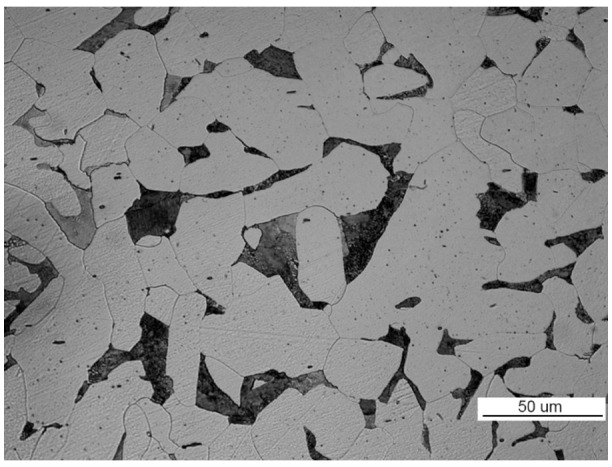
The structure of the steel in part 1 (handy-size bulk carrier 30185 DWT). Optical microscopy.

Such large amounts of impurities significantly reduced the ductile properties of the material, particularly its impact strength. After being etched with an alcoholic solution of nitric acid, the metallographic specimens revealed the ferritic-pearlitic structure of the material from part 1 with an intermittent mesh of precipitated cementite at the ferrite grain boundaries (Figure 45).

**Figure 46:**
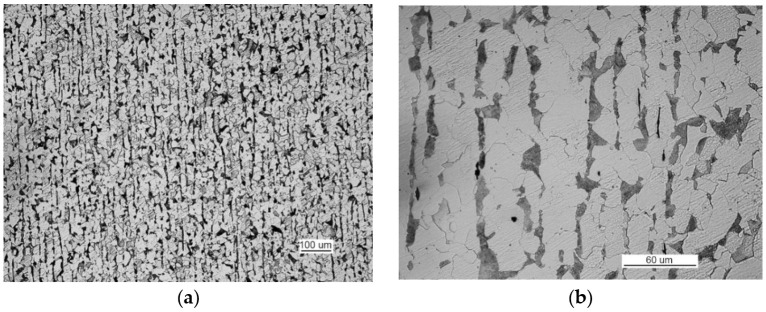
Optical microscopy views (handy-size bulk carrier 30185 DWT): (**a**) the band-like structure of part 2 and (**b**) material structure of part 2 with cementite, silicate, and sulfide precipitates.

The structure of the material in part 2 is shown in Figure 46. The material of part 2 has a ferritic-pearlitic, band-like structure (Figure 46a) with cementite precipitates at grain boundaries, numerous silicates, and rolled sulfides (Figure 46b).

#### 3.4.3. Mechanical Property Tests

A static tensile test was conducted according to PN-EN 10002-1 [59] on φ6 mm samples taken from part 1. The results are shown in Table 8, which corresponds to S275 steel according to PN-EN 10,025 [63].

Charpy impact tests were conducted at −20 °C on samples taken from part 1 perpendicular to its edge, and the results are shown in Table 9. The obtained impact energy values are very low.

#### 3.4.4. Fractography Analysis

**Figure 47:**
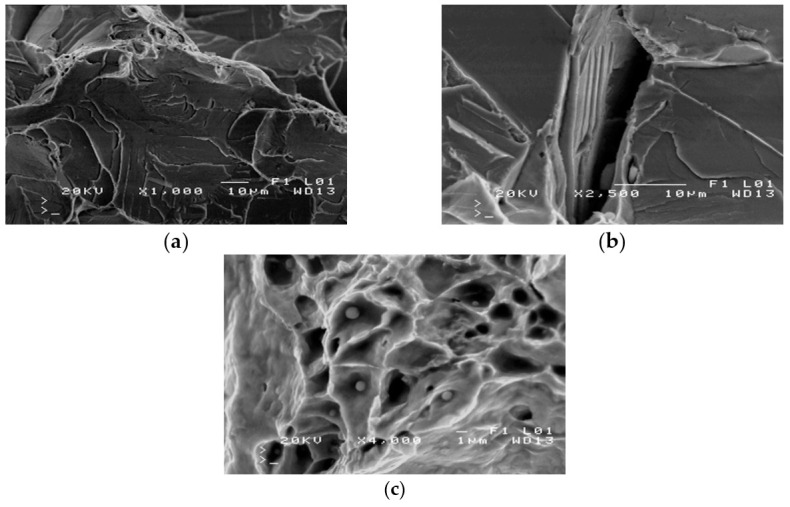
Scanning electron microscope (SEM) views (handy-size bulk carrier 30185 DWT): (**a**) brittle fracture produced after impact testing at −20 °C; (**b**) fracture-based crack that runs through a pearlite grain; and (**c**) a fragment of crater-like ductile fracture (spherical precipitates of Fe and Si oxides are visible at the bottom of craters).

Fractographic analyses were conducted using an SEM with samples obtained from the impact tests. The fractures are shown in Figure 47.

The fractures displayed brittle structures, with cracks running along the cleavage planes to form a distinct river-basin-like structure (Figure 47a). Fracture-based cracks were found (Figure 47b). Small bands revealing plastic strain (Figure 47c) with a crater-like structure are observed locally, with spherical precipitates of iron and silicon oxides visible at the bottom of the craters.

#### 3.4.5. Summary

In the section of the manhole structure, cracking analysis was largely limited by the severely advanced corrosion processes that damaged the fracture surface, making it impossible to directly determine the causes of cracking, the origin, and the course of development.

Crack I, which was initiated in part 2 and expanded across the welds in part 1, was caused by stress in the material and corrosion. The corrosion factor was very important, and the intensity of the corrosion processes was greatly influenced by the purity and homogeneity of the steel used. In the investigated case, both of these factors are sources of concern. The results imply that the applied paint coatings do not sufficiently protect manhole elements because they are easily damaged at the edges, especially with the highly-contaminated steel used in this case. The test pieces are operated under bending load. In contrast to the uniformity of the cross-section subject to a tensile load, where the crack originates in the axis of the item affected by a load, the area most stressed during bending is the outermost layer of the piece, and this is where the crack was initiated. The crack then propagates most intensively in the outermost layer.

Being highly contaminated, the tested steel has a very low impact strength, which results in easy crack initiation due to dynamic loads, especially at low temperatures.

### 3.5. Root Causes Synthesis

In summary, the research results presented in the article and referring to the conducted literature analysis are connected with a number of root causes contributing to the cracking in the structural elements of manholes. The root causes can be categorized according to one of the concepts of Ishikawa diagrams [64] into the following groups: material, method, environment (external factors), operating conditions, and human factor.

Table 10 presents a classification of these groups of causes and their involvement in basic fatigue destruction factors, i.e., material properties, residual stresses, and cyclic load characteristics. The causes contributing to appropriate factors are marked by an X sign. Particular items and their description (cause symbols) are presented in the text following the table.

The material factors related to the material from which the described damaged main deck components were made include the following:

(1) MA1—Technological contamination of the material (uncleaned, unprepared material);
(2) MA2—Contamination resulting from the influence of external factors, e.g., corrosion;
(3) MA3—Susceptibility to residual stresses.

The factors related to the method understood as joining structural elements of the deck by welding, riveting, bolting, etc. include the following:

(1) ME1—Failure to meet technological parameters, including the processes of joining sheets (e.g., welding);
(2) ME2—Occurrence of welding stresses, including residual stresses, in shaping and joining of materials;
(3) ME3—Inadequate calibration of technological devices (machine tools, welders, ultrasonic detectors for tests, etc.);
(4) ME4—Inadequate working conditions in the production plant;
(5) ME5—Inadequate quality control discipline (lack of an appropriate non-destructive testing approach such as ultrasonic testing to access the quality and integrity of welds and a material quality assurance program).

The influence of the environment (external factors, including the atmospheric ones) on the operation object, i.e., the influence of the environment on the ship operation at a specific time and geographical location, includes the following factors:

(1) EX1—Electrochemical interaction of the environment (seawater, water mist, salt, etc.);
(2) EX2—Thermodynamic influence of the environment (water temperature, air temperature, humidity, atmospheric pressure, etc.);
(3) EX3—The influence of hydrometeorological factors (wind force and direction, variable currents, eddies, waves, tides, etc.).

The ship operating conditions associated with cyclic mechanical deformations of the hull during the operation of the ship at sea includes the following factors:

(1) OC1—Effect of shipload (cargo distribution, trim, heel, etc.);
(2) OC2—The influence of the sailing area (sailing in limited and shallow waters).

The human factor, including human interaction during the construction and operation of a ship, concerns the following:

(1) HF1—Lack of appropriate qualifications of the yard’s employees;
(2) HF2—Lack of proper professional preparation of the ship’s service;
(3) HF3—Lack of decision and negligence by the shipyard‘s management and ship’s personnel;
(4) HF4—Negligence during quality control in the shipyard;
(5) HF5—Insufficient technological discipline among the shipyard’s employees and ship’s crew;
(6) HF6—Psychophysical disability of the shipyard’s employees and ship’s crew (illness, exhaustion, influence of psychoactive substances);
(7) HF7—Time pressure on the shipyard and shipowner’s employees.

The synthesis of different root causes presented in Table 10 proves that the most significant influence is connected with material properties (80% of all fatigue damage contribution factors). Next, are residual stresses and cyclic load characteristics, which account for 70% and 50%, respectively.

## 4. Conclusions

In all examined cases, the cracking in the structural elements of manholes was caused by the low quality of the steel used. Local strain concentration—caused by various kinds of notches—was not relieved by local plastic strain. This property and the low ductility of the steel, especially when kept at low temperatures, caused strain to be relieved by cracking. The effect of stress within deck plates under extreme loads in the hull in port or at sea can also cause cracking. The examined cracks occurred due to the manhole structure strain under the ship operating conditions, which exceeded the strength of the steel used, indicating structural defects in the vessel.

This confirms the hypothesis proposed in the introduction that the main decks crack around manholes because of the very low-quality welded joints and because the manholes were made from poor-quality steel that contained large amounts of non-metallic impurities. The prevention of similar cracks in the future requires:

(1) For the structural parts of the manhole, using steel with suitable mechanical properties, including an impact energy corresponding to at least marking J2; use steel tested for defects in materials that can initiate cracks;
(2) Developing the right technology for welding work and closely monitoring compliance with respective regulations;
(3) Applying non-destructive testing (e.g., eddy-current, magnetic-particle, liquid penetrant, radiographic, ultrasonic, or acoustic emission) to access the quality and integrity of welds and material quality assurance program;
(4) Modifying the ship’s design in the context of variable mechanical loads carried by the hull.

Further research on the presented topic of ship machinery destruction in terms of corrosion fatigue and complex load situations might include a combination of the material analysis as presented in this paper with the issues of residual stresses resulting from welding. Moreover, the finite element method may be involved to receive a holistic approach to the ship’s operational safety and reliability.

## Figures and Tables

**Table 1:** Basic data of ships whose manholes were analyzed.

No.	DWT	Year of Manufacturing	Length (m)	Beam (m)	Working Time (Days)
1	30,182	2012	189	24	1206
2	30,206	2012	190	23	1200
3	30,210	2011	190	24	1412
4	30,185	2011	189	24	1606

**Table 2:** The results of the impact strength tests of the samples taken from handy-size bulk carrier 30182 DWT.

Test Temperature (°C)	Sample No./Test No.	Impact Energy KV 100 (J)
0	2/1	Above 100
−20	2/1	44
−20	2/2	91
−20	1/1	21
−20	1/2	76
−40	2/1	11
−40	1/1	10
−40	1/2	13

**Table 3:** The chemical composition of the sample taken from the handy-size bulk carrier 30206 DWT. X-ray microanalysis.

Element	Content (% m/m)
C (carbon)	0.65
O (oxygen)	31.14
Na (sodium)	1.43
Mg (magnesium)	1.31
Al (aluminum)	3.65
Si (silicon)	12.4
K (potassium)	3.27
Ca (calcium)	4.55
Ti (titanium)	21.74
Cr (chromium)	0.1
Mn (manganese)	8.29
Fe (iron)	10.40

**Table 4:** Results of the static tensile tests of samples from the handy-size bulk carrier 30206 DWT.

Sample No.	The Diameter of the Measured Part (mm)	Ultimate Tensile Strength *R_m_* (MPa)	Elongation at Break *A* (%)
1	6	465	32
2	6	458	35

**Table 5:** Results of the impact strength test of samples from the handy-size bulk carrier 30206 DWT.

Sample No.	Impact Energy KV^0^100 (J)
1	>98
2	54
3	83

**Table 6:** Results of the static tensile tests of samples from the handy-size bulk carrier 30210 DWT.

Sample No.	Yield Point *R*_e_ (MPa)	Ultimate Tensile Strength *R*_m_ (MPa)	Elongation after a Break *A* (%)	Waist Formation *Z* (%)
1	277	416	23	62
2	330	458	34	58

**Table 7:** Results of the impact strength test of samples from the handy-size bulk carrier 30210 DWT.

Sample No.	Impact Energy KV^−20^ (kGm)	Impact Energy KV^−20^ (J)
1	0.5	4.9
2	0.9	8.8
3	0.7	6.9

**Table 8:** Results of the static tensile test of samples from the handy-size bulk carrier 30185 DWT.

Sample No.	Yield Point *R*_e_ (MPa)	Ultimate Tensile Strength *R*_m_ (MPa)	Elongation after a Break *A* (%)	Waist Formation *Z* (%)
1	263	416	34	64
2	256	416	40	58
3	284	430	28	58

**Table 9:** Results of the Charpy impact strength tests of samples from the handy-size bulk carrier 30185 DWT.

Sample No.	Impact Energy KV^–20^ (kGm)	Impact Energy KV^–20^ (J)
1	1.2	11.8
2	0.9	8.8
3	0.7	6.9

**Table 10:** List of the factors affecting cracks of manholes.

Causes Group	Cause Symbol	Fatigue Damage Factors		
		Material Properties	Residual Stresses	Cyclic Load Characteristics
Material	MA1	X		
	MA2	X		
	MA3	X	X	
Method	ME1	X	X	
	ME2		X	
	ME3	X	X	
	ME4	X	X	
	ME5	X	X	
Environment	EX1	X	X	X
	EX2	X	X	X
	EX3			X
Operating conditions	OC1			X
	OC2			X
Human factor	HF1	X	X	
	HF2	X		X
	HF3	X	X	X
	HF4	X	X	
	HF5	X	X	X
	HF6	X	X	X
	HF7	X	X	X

## Data Availability

All results are provided in the paper.

## Nomenclature

*A*	Elongation after a break
AA5b	Material structure pattern according to standard [57].
DWT	Deadweight tonnage
FSA	Formal Safety Assessment
IMO	International Maritime Organisation
KN5b	Material structure pattern according to standard [57].
*KV^XX^*	V-notched Charpy impact strength test absorbed energy at the temperature *XX.*
PN	Polish Standard
SEM	Scanning Electron Microscope
TP5a	Material structure pattern according to standard [57].
*R_e_*	Yield point
*R_m_*	Ultimate tensile strength
*Z*	Waist formation
